# Inetetamab triggers cardiotoxicity through its interaction with apoptosis, oxidative stress and autophagy pathways

**DOI:** 10.1038/s41598-025-02125-5

**Published:** 2025-07-01

**Authors:** Weiqun Wang, Hongliang Zhang, Yikun Qu, Yue Li, Peng Peng, Chengbo Lu, Xinyue Zhao, Ziteng Cai, Chaonan Peng, Xiaoli Guo, Yuxin Guo, Jie Li, Xuebin Li, Linlin Jia, Guangyuan Yang

**Affiliations:** 1 The Basic Medical College, Jiamusi University, Jiamusi, 154007 Heilongjiang China; 2 State Key Laboratory of Neurology and Oncology Drug Development, Nanjing, 210018 Jiangsu China; 3 Department of Cardiovascular Medicine, The First Affiliated Hospital, Jiamusi University, Jiamusi, 154007 Heilongjiang China; 4 Zhangzhou Health Vocational College, Zhangzhou, 363000 Fujian China; 5 First Affiliated Hospital, State Key Laboratory of Neurology and Oncology Drug Development, and Basic Medical College, Jiamusi University, No.258 of Xuefu street, Jiamusi, 154002 Heilongjiang People’s Republic of China

**Keywords:** Inetetamab, Cardiotoxicity, Oxidative stress, Apoptosis, Autophagy, Biochemistry, Cancer, Cell biology, Physiology, Toxicology

## Abstract

Inetetamab, a trastuzumab biosimilar, has obtained approval for the treatment of HER2-positive advanced metastatic breast cancer. Despite studies indicating its cardiotoxic properties, the intricate mechanisms underlying its toxicity remain elusive. In the present study, we demonstrated in vitro that exogenous administration of Inetetamab triggered injury in H9c2 myocardial cells, characterized by a significant decrease in cell viability and noticeable morphological changes. Furthermore, exogenous exposure to Inetetamab elicited a series of toxic effects within H9c2 cells, notably promoting apoptosis and autophagy, diminishing the mitochondrial membrane potential (DCm), and elevating intracellular ROS production. Concurrently, there was a notable decrease in intracellular MDA generation, accompanied by disruption of the GSH/GSSG balance. In vivo mouse model, Inetetamab administration prominently induced cardiomyocyte injury, characterized by the pathological change in the myocardial tissue and a marked elevation in serum levels of creatine kinase isoenzyme (CK-MB), brain natriuretic peptide (BNP) and cardiac troponin I (cTnI). Similarly, Inetetamab administration also initiated cardiomyocyte apoptosis and oxidative stress injury in vivo. Furthermore, Inetetamab administration significantly modulated the expression of apoptosis- and autophagy-related proteins both in vivo and in vitro. Our study highlights Inetetamab induces cardiotoxicity by affecting apoptosis, oxidative stress and autophagy.

**Supplementary Information:**

The online version contains supplementary material available at 10.1038/s41598-025-02125-5.

## Introduction

Breast cancer remains a life-threatening disease and is the second most common cause of mortality among women worldwide. Since 2004, the prevalence of breast cancer has steadily risen, increasing at a rate of 0.5% annually^1,2^. Surgical resection, chemotherapy, radiation, endocrine therapy, small molecule inhibitor therapy, immunotherapy, gene therapy, and the integration of these methods are employed in the treatment of breast cancer^3,4^. Despite significant advancements in therapeutic modalities for breast cancer, the heterogeneity of the disease, medication resistance, severe adverse effects and poor selectivity continue to pose significant challenges in reducing recurrence and mortality. The subtype of breast cancer is the main factor determining systemic treatment. Based on molecular markers, breast cancer can be categorized into three primary subtypes, each exhibiting distinct risk profiles: estrogen or progesterone receptor-positive (HR+), human epidermal growth factor receptor 2-positive (HER2+) and triple-negative (TN) breast cancer^5,6^. The HER2 + subtype is present in approximately 20% of breast cancer patients, characterized by the amplification of the HER2 gene or the overexpression of its protein. HER2 is strongly associated with the aggressiveness and prognosis of tumors, rendering it a pivotal target for the development of anticancer drugs, with these targeted therapies significantly improving survival outcomes for patients with HER2 + breast cancer^5,6^.

Inetetamab, an pioneering humanized monoclonal antibody specifically designed to target HER2, was developed in China and received recommendation by the National Medical Products Administration (NMPA) of China for the treatment of HER2 + metastatic breast cancer in 2020^7^. The Fc region of Inetetamab can be modified by amino acid, thereby enhancing its ability to elicit antibody-dependent cell-mediated cytotoxicity (ADCC). Recent evidence has demonstrated that Inetetamab, administered either as monotherapy or in combination with other treatments or drugs exhibits remarkable efficacy and synergistic anti-cancer effects in clinical trials^7–9^. Nevertheless, it is acknowledged that cardiovascular events may be a potential complication associated with anti-HER2 therapy, particularly when anti-HER2 drugs are administered in combination with chemotherapy agents such as Doxorubicin and Epirubicin, which have been found to increase the risk of cardiotoxicity among patients^10,11^. According to China’s stringent pharmaceutical service guidelines for antibody anticancer drugs, an assessment of the patient’s left ventricular function must be conducted before starting Inetetamab treatment and during the treatment period^12^. For patients with metastatic HER2-positive breast cancer receiving Inetetamab treatment, if a significant decline in left ventricular function is detected, the current treatment regimen should be immediately discontinued^12^. However, the precise mechanism responsible for Inetetamab-induced cardiotoxicity remains elusive.

The primary aim of this study is to determine whether Inetetamab possesses the capacity to elicit cardiotoxicity at both cellular and animal levels, and to uncover the underlying molecular mechanisms that are responsible for Inetetamab-induced myocardial injury, with a particular focus on exploring the complex interplay among apoptosis, oxidative stress and autophagy. This study offered a novel perspective that may aid in the rational utilization of Inetetamab for the management of HER2 + metastatic breast cancer.

## Materials and methods

### Cell line and cell culture

H9c2 rat cardiomyocyte line, obtained from the American Type Culture Collection (ATCC; Manassas, VA, USA), was cultured in high-glucose Dulbecco’s Modified Eagle Medium (DMEM; Gibco, Rockville, MD, USA) supplemented with 10% fetal bovine serum (FBS; Gibco, Rockville, MD, USA). Cells were maintained at 37 °C in a humidified atmosphere containing 5% CO_2_.

### Treatment of cells with Inetetamab and CCK-8 assay

H9c2 cells (3 × 10^3^ cells per well) were seeded into 96-well plates containing 100 µl of medium supplemented with 10% fetal bovine serum (FBS). Once the cells had reached a growth density of approximately 70–80%, the medium was replaced with fresh medium containing various concentrations of Inetetamab (cat. no S20200012; Sansheng Guojian Pharmaceutical, China). Each experimental group consisted of seven replicate wells. After 48 h of culture, cell viability was estimated using a CCK-8 assay kit (cat. no AR119; Boster, China) in accordance with the manufacturer’s instructions. Based on the results, the viability of the cells andthe half maximal inhibitory concentration (IC50) were calculated.

### TUNEL and DCm assays

After 48 h of incubation with various concentrations of Inetetamab, the apoptosis rate and the mitochondrial membrane potential (DCm) within H9c2 cells were respectively assessed using a TUNEL assay kit (cat. no C1088; Beyotime, China) and a JC-1 DCm assay kit (cat. no C2003S; Beyotime, China), following the manufacturer’s instructions. The experimental results were observed and analyzed using a laser scanning confocal microscope (Olympus, Japan).

### Autophagy assay

After 48 h of incubation with various concentrations of Inetetamab, H9c2 cells were collected and subsequently rinsed with PBS. Following this, the cells were resuspended in an appropriate amount of acridine orange (AO) staining buffer (cat. no CA1143; Solarbio, China). After adjusting the cell count to 10^6^/ml, 95 µl of the cell suspension was mixed with 5 µl of AO staining solution. The mixture was then incubated in a dark environment at room temperature for 15 min. The mixed cell suspension was dispensed onto glass slides, which were subsequently covered with coverslips. The intensity of fluorescence emitted from the samples was observed and analyzed under a laser confocal microscope (Olympus, Japan).

### ROS detection

After 48 h of treatment with Inetetamab, the levels of reactive oxygen species (ROS) in H9c2 cells were quantified utilizing a ROS detection kit (cat. no S0033S; Beyotime, China), following the manufacturer’s instructions. ImageJ software was used to quantitatively analyze the intensity of green fluorescence, which serves as an indicator of ROS levels.

### Animals and their breeding

A total of 30 female SPF ICR mice, weighing between 18 and 22 g, were purchased from Heilongjiang Yuheng Veterinary Technology Service Co., Ltd. [License No. SCXK (Hei) 2019-001]. These mice were housed in a temperature-controlled environment with humidity levels maintained within the range of 45–65%. They were subjected to a standard 12-hour light-dark cycle and provided with ad libitum access to food and water. The authors complied with the ARRIVE guidelines. All methods were carried out in accordance with relevant guidelines and regulations, and all studies and protocols were granted approval by the Animal Ethics Committee of Jiamusi University (Approval No. 20220025).

### The establishment of a cardiotoxicity mice model induced by Inetetamab

After a 7-day period of acclimatization feeding, the 30 mice were evenly divided into three groups based on our cell study and preliminary experiments: a Control (0 mg/kg) group, a 4 mg/kg Inetetamab group and an 8 mg/kg Inetetamab group. Each group received either saline (for Control group) or Inetetamab dissolved in saline at specified doses once weekly via intraperitoneal injection, in accordance with established protocols^13,14^. On day 28 post-injection, the mice were euthanized using CO2 asphyxiation, and subsequently, serum and myocardial tissue samples were harvested for further experimental analysis.

### Cardiotoxicity biomarkers detection

The serum levels of creatine kinase isoenzyme (CK-MB), brain natriuretic peptide (BNP) and cardiac troponin (cTnI) were determined using ELISA kits, following the respective protocols provided by the manufacturer (cat. no MM-43703M1, MM-0060M1 and MM0427M1; Beyotime, China).

### Hematoxylin and eosin (HE) staining

The hearts were harvested and fixed in a 0.1 mol/L phosphate-buffered saline (PBS) solution containing 4% paraformaldehyde for 24 h. Following fixation, the hearts underwent a series of processes including dehydration, embedding and slicing. The sections, which were 4–5 micrometers in thickness, were stained with hematoxylin and eosin (HE) for subsequent observation under an optical microscope.

### Hoechst 33,342 staining

Apoptosis of cardiomyocytes in mice was investigated by staining heart sections with a Hoechst 33,342 staining kit (catalog number C0031; Solarbio, China), following the manufacturer’s instructions. The experimental results were then observed under a fluorescence microscope (Nikon E200MV, Japan).

### Immunohistochemistry assay

After undergoing the standard procedures of deparaffinization, hydration, blocking and antigen retrieval, the heart sections were incubated overnight at 4 °C with a primary antibody specifically targeting either Bax (diluted 1:50; cat. no CY5059; Abways, China) or Caspase-3 (diluted 1:50; cat. no AF6311; affinity, China). Subsequently, a SABC kit (cat. no SA1028; Boster, China) along with a DAB substrate kit (cat. no AR1027; Boster, China) was employed for the detection of proteins via colorimetric visualization.

### GSH/GSSG ratio, MDA content and SOD activity detection

Supernatants from myocardial tissues and H9c2 cells were collected, and specific ELISA kits (cat. No S0053, S0131S, and S0101S; Beyotime, China) were used to assess GSH/GSSG ratio, MDA content, and SOD activity, all in accordance with the manufacturer’s instructions.

### Western blotting

Total proteins were extracted from myocardial tissues or H9c2 cells using RIPA buffer. The proteins were separated by 10% SDS-PAGE and subsequently transferred onto polyvinylidene difluoride (PVDF) membranes. After blocking with milk, the membranes were incubated overnight with primary antibodies, including Caspase-3 (diluted 1:1000; cat. no AF6311; affinity, China), Bcl-2 (diluted 1:1000; cat. no CY5032; Abways, China), Bax (diluted 1:000; cat. no CY5059; Abways, China), P62 (diluted 1:000; cat. no 21S51; Boster, China) and Beclin1 (diluted 1:000; cat. no CY5092; Abways, China). Following incubation with secondary antibodies (Boster, China) and ECL reagent, the protein bands were visualized using an automatic chemiluminescence imaging analysis system (Tanon, China).

### Statistical analysis

SPSS 26.0 software was utilized for statistical analysis, including one-way analysis of variance (ANOVA) among multiple groups. The data were presented as mean ± standard deviation, and a P-value < 0.05 was considered statistically significant.

## Results

### Inetetamab reduced the viability of H9c2 cells and caused changes in theirmorphology

**Fig. 1:**
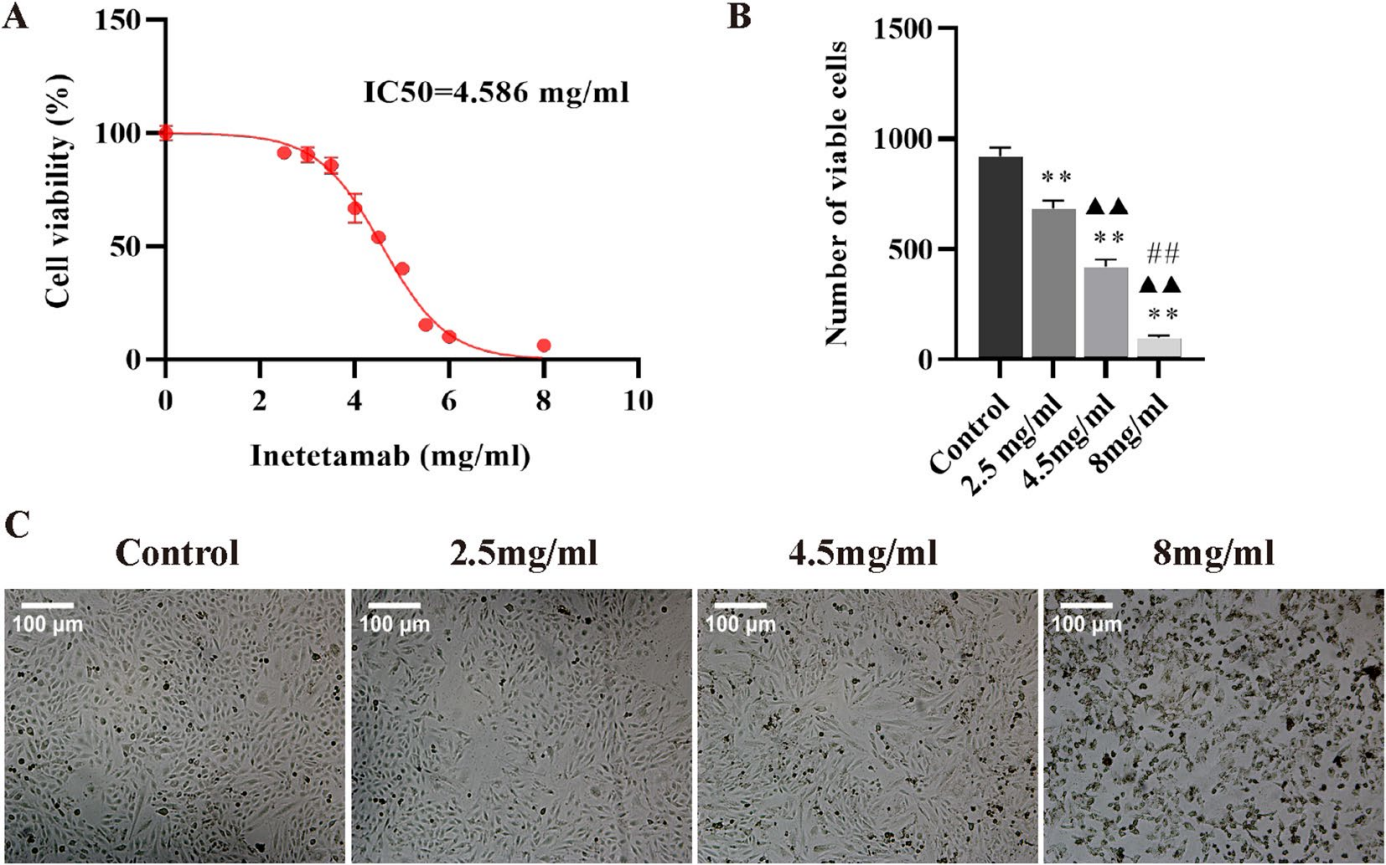
Inetetamab reduced cell viability and induced morphological changes of the H9c2 cells in a dose-dependent manner. (**A**) An IC50 analysis of Inetetamab’s impact on the H9c2 cells was determined utilizing CCK-8 assay (*n* = 5). (**B**) CCK-8 assay was employed to quantify the viable H9c2 cell count following treatment with varying concentrations of Inetetamab (*n* = 5). (**C**) Representative microscopic images showed the morphological changes in the H9c2 cells following treatment with varying concentrations of Inetetamab. **P < 0.01 vs. Control, ^▲▲^*P* < 0.01 vs. 2.5 mg/ml, ^##^*P* < 0.01 vs. 4.5 mg/ml.

CCK-8 assay results indicated a dose-dependent decrease in the viability of cells following Inetetamab treatment, with an IC50 value of 4.586 mg/ml (Fig. 1A). Therefore, different concentrations of Inetetamab were chosen for further experimentation, including lowest concentration of 2.5 mg/ml, nearly IC50 concentration of 4.5 mg/ml and highest concentration of 8 mg/ml. Subsequent experiments demonstrated that Inetetamab exhibits a pronounced inhibitory effect on the activity of H9c2 cells in a dose-dependent fashion (Fig. 1B). Furthermore, microscopic observations revealed a significant reduction in the number of H9c2 cells as Inetetamab concentration increased from 2.5 mg/ml to 8 mg/ml, passing through 4.5 mg/ml, when compared to Control (0 mg/ml) cells. At the lowest concentration of 2.5 mg/ml, noticeable morphological alterations were observed in H9c2 cells. At 4.5 mg/ml, some cells exhibited signs of atrophy, indicating early cellular stress. Notably, at the highest concentration of 8 mg/ml, significant morphological changes were observed, characterized by a dry and rough plasma membrane, strongly suggesting extensive cell damage. (Fig. 1C).

### Inetetamab induced apoptosis, mitochondrial dysfunction and autophagy in H9c2 cells

**Fig. 2:**
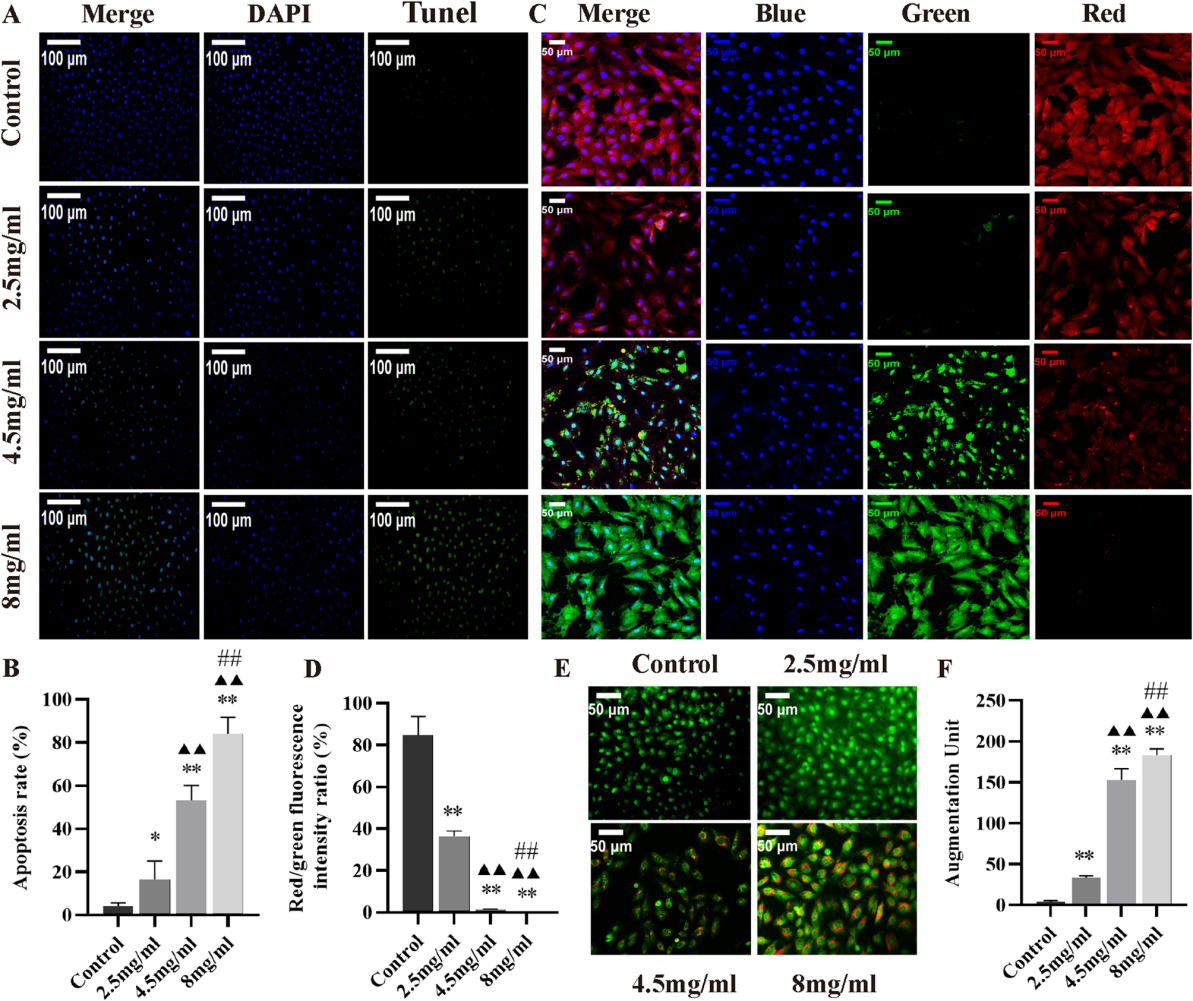
Inetetamab triggered apoptosis, mitochondrial and dysfunction autophagy of the H9c2 cells in a dose-dependant manner. (**A**) TUNEL staining confirmed that Inetetamab enhanced apoptosis of the H9c2 cells. (**B**) The quantification of apoptosis rates was determined in the H9c2 cells from A (*n* = 5). (**C**) JC-1 staining confirmed that Inetetamab decreased the mitochondrial membrane potential of the H9c2 cells. (**D**) The mitochondrial membrane potential of the H9c2 cells was quantified using a fluorescence intensity ratio between red and green emissions from C (*n* = 5). (**E**) AO staining confirmed that Inetetamab enhanced autophagy of the H9c2 cells. (**F**) The quantification of autophagy was determined in the H9c2 cells from E (*n* = 5). ^*^P < 0.05, ^**^P < 0.01 vs. Control, ^▲▲^*P* < 0.01 vs. 2.5 mg/ml, ^##^*P* < 0.01 vs. 4.5 mg/ml.

TUNEL staining results showed that H9c2 cells treated with 2.5 mg/ml, 4.5 mg/ml and 8 mg/ml of Inetetamab exhibited significantly increased levels of apoptosis compared to Control cells (Fig. 2A). Additionally, the apoptosis rate demonstrated a dose-dependent manner in response to Inetetamab treatment (Fig. 2B). Subsequently, we conducted DCm testing, which revealed that different concentrations of Inetetamab significantly reduced DCm in a dose-dependent manner, as confirmed by JC-1 staining in H9c2 cells (Fig. 2C, D). AO staining experiments revealed that, in H9c2 cells treated with Inetetamab, the red fluorescence intensity of autophagosomes stained by AO significantly increased in a dose-dependent manner, indicating that Inetetamab induces autophagy in these cells (Fig. 2E, F).

### Inetetamab augmented oxidative stress injury in H9c2 cells

**Fig. 3:**
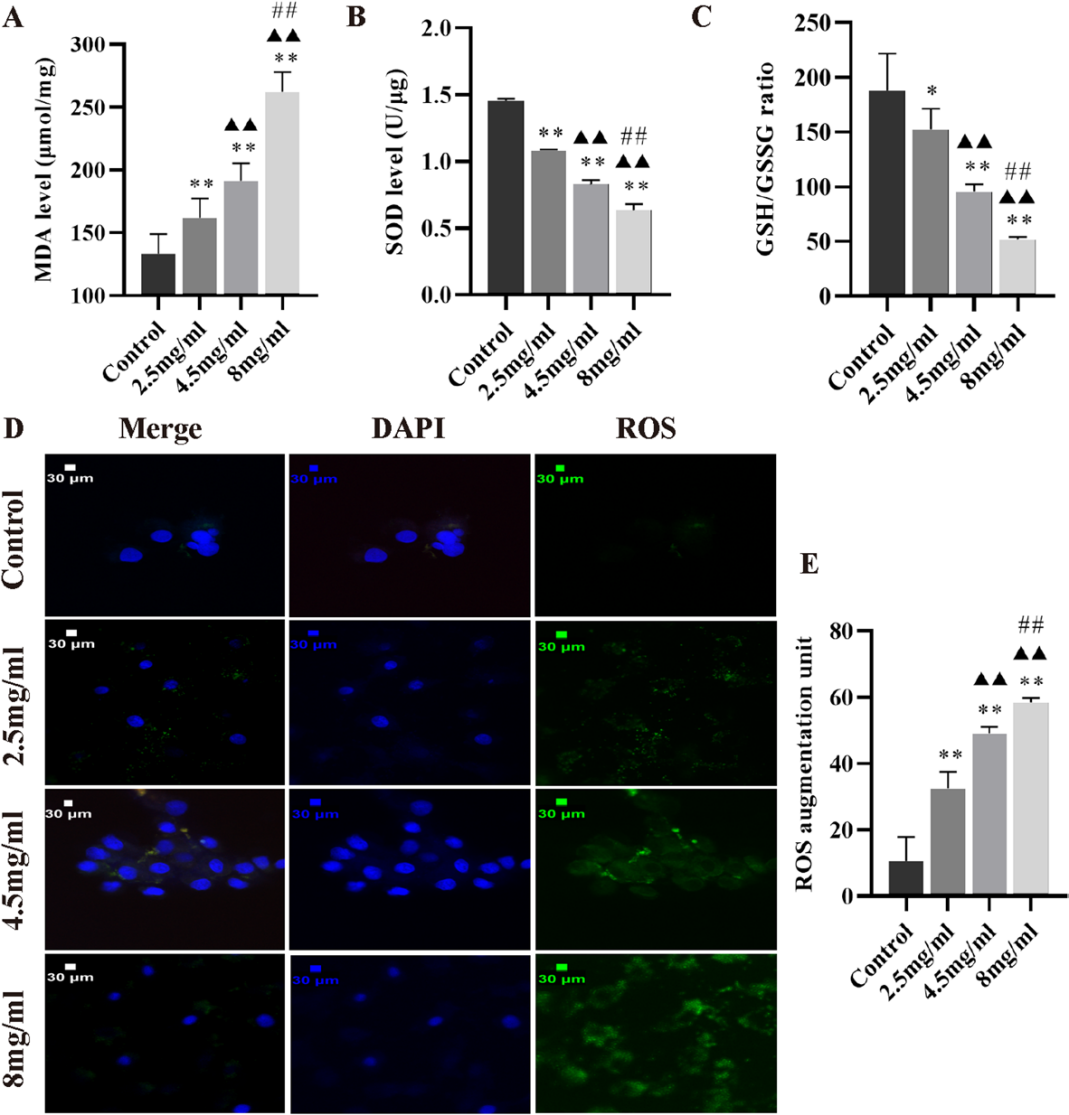
Inetetamab enhanced oxidative stress injury of the H9c2 cells in a dose-dependant manner. (**A**) Inetetamab enhanced MDA levels in the H9c2 cells (*n* = 5). (**B**) Inetetamab reduced SOD levels in the H9c2 cells (*n* = 5). (**C**) Inetetamab reduced the GSH/GSSH ratio in the H9c2 cells (*n* = 5). (**D**) Inetetamab enhanced ROS levels in the H9c2 cells. (**E**) The quantification of ROS levels was determined in the H9c2 cells from D (*n* = 5). ^*^P < 0.05, ^**^P < 0.01 vs. Control, ^▲▲^*P* < 0.01 vs. 2.5 mg/ml, ^##^*P* < 0.01 vs. 4.5 mg/ml.

Subsequently, our investigation into oxidative stress indicators in H9c2 cells revealed that Inetetamab dose-dependently increases MDA levels (Fig. 3A), while concurrently decreasing SOD activity (Fig. 3B) and the GSH/GSSG ratio (Fig. 3C). Additionally, the level of reactive oxygen species (ROS) produced by H9c2 cells exhibited a dose-dependent increase as the concentrations of Inetetamab increased (Fig. 3D, E). These findings imply that Inetetamab has the potential to induce oxidative stress injury in cardiomyocytes in vitro.

### Inetetamab regulated the expression of apoptosis/autophagy-associated proteins in H9c2 cells

**Fig. 4:**
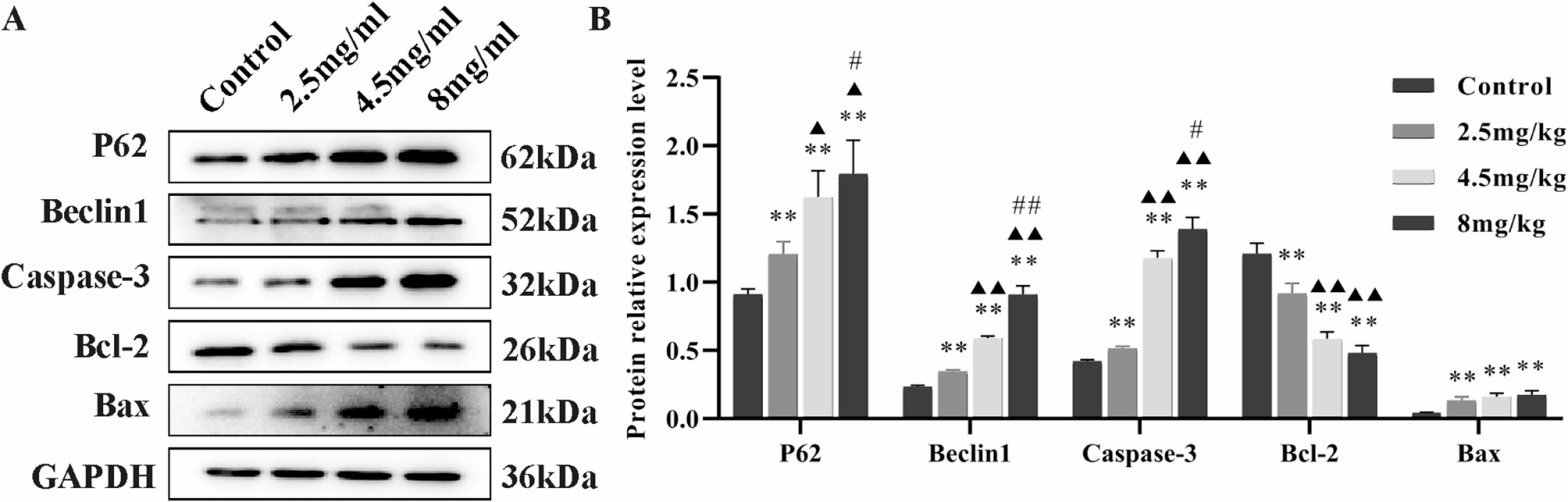
Inetetamab regulated the expression levels of apoptosis- and autophagy-related proteins of the H9c2 cells in a dose-dependant manner. (**A**) Inetetamab modulated the expression levels of P62, Beclin 1, Caspase-3, Bcl-2 and Bax proteins in the H9c2 cells. (**B**) The quantification of the aforementioned proteins was conducted in the H9c2 cells from A (*n* = 3). **P < 0.01 vs. Control, ^▲▲^*P* < 0.01 vs. 2.5 mg/ml, ^##^*P* < 0.01 vs. 4.5 mg/ml.

To elucidate the potential mechanism underlying Inetetamab’s effect on H9c2 cells, we conducted Western blotting experiments to investigate the expression levels of proteins associated with apoptosis/autophagy following Inetetamab treatment. The results showed that Inetetamab treatment significantly upregulated the expression of P62, Beclin-1, Caspase-3 and Bax proteins, while downregulating Bcl-2 protein levels, in a dose-dependent manner (Fig. 4A, B).

### Intraperitoneal administration of Inetetamab induced cardiotoxicity in a mouse model

**Fig. 5:**
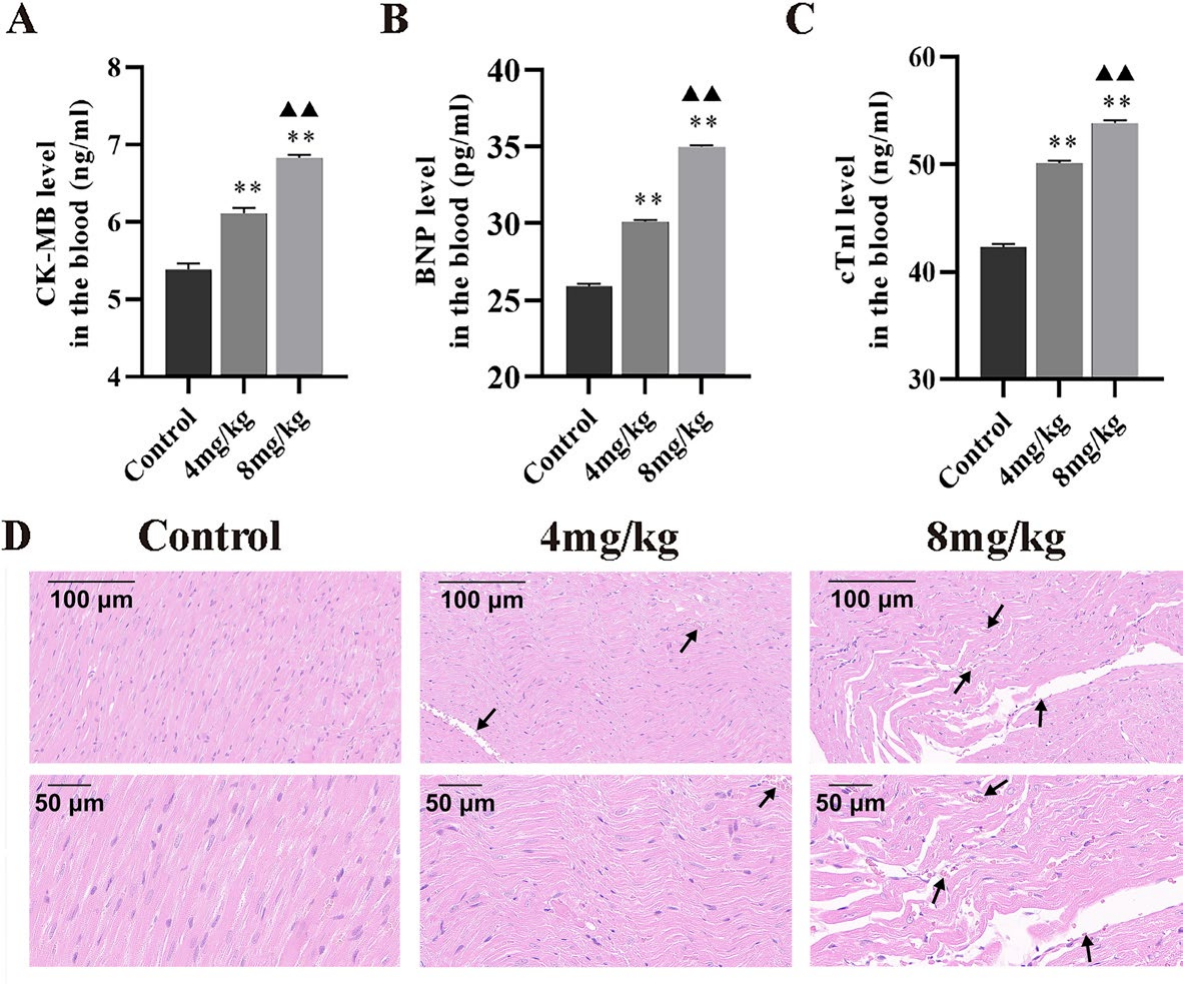
Inetetamab triggered cardiotoxicity in mice. (**A**) The quantification of CK-MB levels was determined in blood samples of mice (*n* = 5). (**B**) The quantification of BNP levels was determined in blood samples of mice (*n* = 5). (**C**) The quantification of cTnl levels was determined in blood samples of mice (*n* = 5). (**D**) HE staining demonstrated that Inetetamab administration markedly induced evident myocardial tissue damage. ^**^P < 0.01 vs. Control, ^▲▲^*P* < 0.01 vs. 4 mg/ml.

In the vivo experiments, we first assessed the effects of Inetetamab on cardiotoxicity using parameters related to heart function. Our results indicated that intraperitoneal administration of Inetetamab led to a dose-dependent elevation in serum levels of CK-MB (Fig. 5A), BNP (Fig. 5B) and cTnl (Fig. 5C) in mice. Furthermore, histological analysis using HE staining revealed that the striations of myocardial fibers are blurred or even absent; the cells exhibit a disordered, wavy arrangement with nuclei of varying sizes. Additionally, there is dilation and congestion of blood vessels in the myocardial interstitium (Fig. 5D).

### Intraperitoneal administration of Inetetamab elicitd myocardial injury in vivo through apoptosis and autophagy pathways

**Fig. 6:**
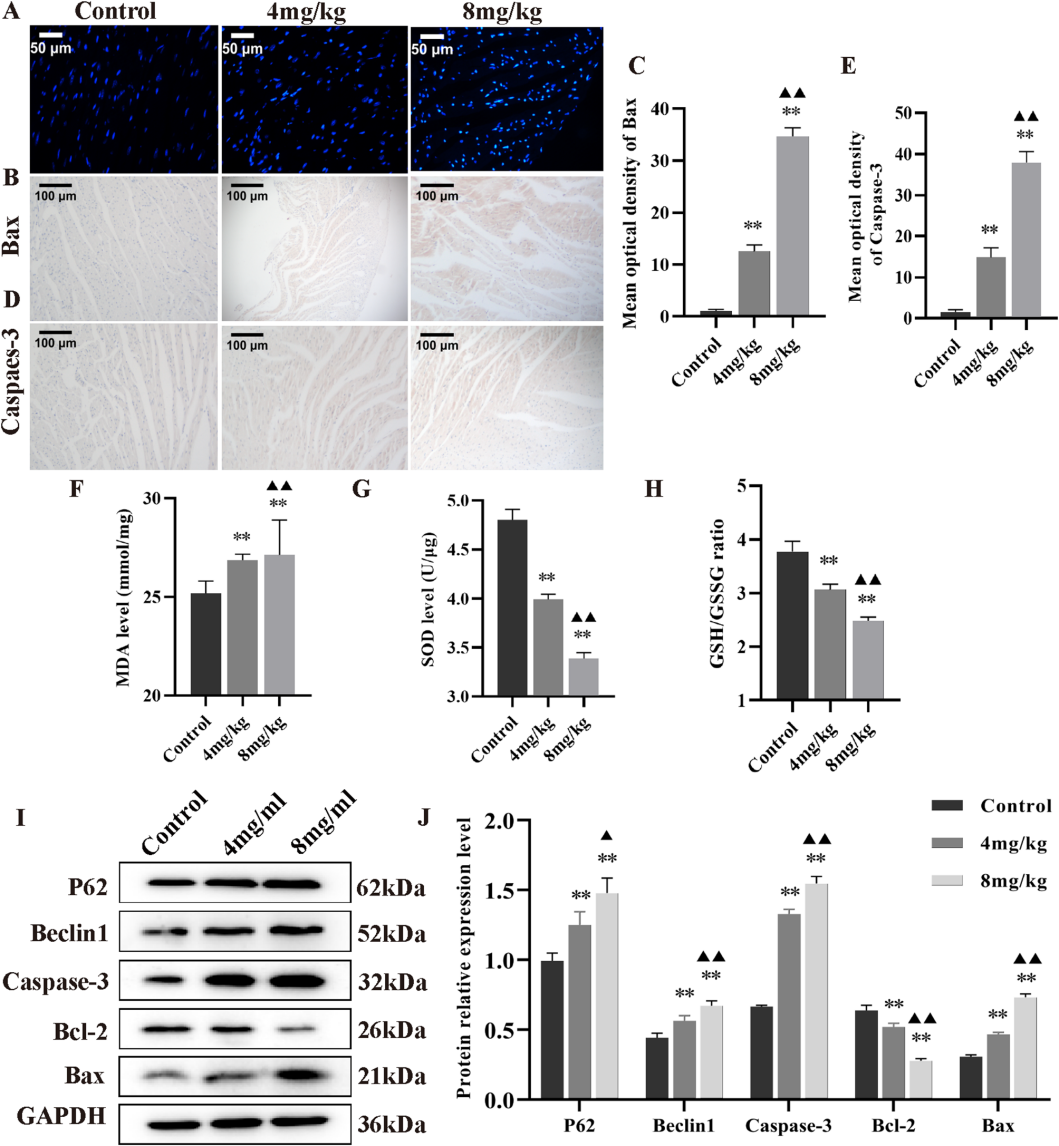
Inetetamab induced myocardial injury in mice through the enhancement of apoptosis and autophagy in a dose-dependent manner. (**A**) Hochest 33,342 staining showed that Inetetamab enhanced myocardial cell apoptosis. (**B**,**D**) Immunohistochemistry analysis demonstrated that Inetetamab upregulated the expression levels of Bax and Caspase-3 proteins in the myocardial tissues. (**C**,**E**) The quantification of Bax and Caspase-3 levels was determined from B and D (*n* = 5). (**F**) Inetetamab enhanced MDA levels in the myocardial tissues (*n* = 5). (**G**) Inetetamab reduced SOD levels in the myocardial tissues (*n* = 5). (**H**) Inetetamab reduced the GSH/GSSH ratio in the myocardial tissues (*n* = 5). (**I**) Inetetamab modulated the expression levels of P62, Beclin 1, Caspase-3, Bcl-2 and Bax proteins in the myocardial tissues. (**J**) The quantification of the aforementioned proteins was conducted from I (*n* = 3). ^**^P < 0.01 vs. Control, ^▲▲^*P* < 0.01 vs. 4 mg/ml.

Finally, we conducted further studies to investigate the potential molecular mechanism of Inetetamab in vivo. Hochest 33,342 staining revealed a significant increase in myocardial cell apoptosis following intraperitoneal administration of Inetetamab (Fig. 6A). Immunohistochemical staining analysis demonstrated that the expression of Bax and Caspase-3 proteins was significantly upregulated in myocardial tissue after intraperitoneal administration of Inetetamab (Fig. 6B–E). Furthermore, consistent with the findings from our cell experiments, ELISA and Western blotting assays revealed that Inetetamab in vivo exhibits a dose-dependent capacity to alter oxidative stress indicators (Fig. 6F–H) and modulate the expression of proteins associated with apoptosis and autophagy (Fig. 6I, J).

## Discussion

Drugs developed utilizing cancer-specific antibodies represent one of the most successful and promising therapeutic strategies in contemporary medicine, as demonstrated by their ability to significantly enhance survival outcomes for cancer patients^15^. Anticancer antibody drugs exert their therapeutic effects through binding to specific antigens on the surface of cancer cells, resulting in a range of actions including serving as agonists or antagonists within cell signaling pathways, inhibiting natural growth and survival signals, or enhancing signals that induce cancer cell death^16^. Inetetamab stands as the pioneering and innovative humanized anti-HER2 monoclonal antibody drug developed and commercialized in China by Sansheng Pharmaceutical Co., Ltd. Accumulating evidence indicated that Inetetamab, whether utilized alone or in combination with other chemotherapeutic drugs, may represent the most efficacious treatment option for patients with HER2 + metastatic breast cancer^7,8^. Although Inetetamab exhibits notable efficacy in the treatment of breast cancer, its clinical application is constrained by potential cardiotoxic side effects that are similar to trastuzumab, the pioneering monoclonal antibody targeting HER2.

It is widely acknowledged that the heart possesses relatively inadequate antioxidant defense capabilities, which significantly increases its susceptibility to oxidative stress-induced damage^17^. Oxidative stress is a detrimental condition that emerges as a result of an imbalance between oxidants and antioxidants, leading to excessive accumulation of reactive oxygen species (ROS), notably including highly reactive hydroxyl radicals. This ultimately results in degradation of phospholipids in the lipid bilayer of membranes, which causes a series of detrimental effects, including damage to membrane integrity and membrane dysfunction^18,19^. A great deal of evidence has suggested that oxidative stress plays a pivotal role in the cardiotoxicity resulting from antitumor drugs, which directly and indirectly participates in their toxic responses^20^. For example, it has been suggested that Doxorubicin has the ability to increase absorption of oxygen and generation of diverse types of ROS, resulting in oxidative stress^21^. ROS production induced by Doxorubicin can initiate pathological sarcoplasmic reticulum (SR) calcium leakage, DNA damage and disruption of autophagic flux, ultimately leading to lipid peroxidation-mediated ferroptosis and various other forms of regulated cell death^22^. Moreover, it has been verified that reducing oxidative stress in cardiomyocytes alleviates the cardiotoxicity triggered by anti-tumor drugs, specifically by regulating the levels of ROS, MDA, SOD and GSH^23^. In certain clinical trials, simultaneous administration of antioxidants along with chemotherapy drugs has exhibited a protective effect against cardiotoxicity caused by oxidative stress^19^. In the current study, we demonstrated that Inetetamab elevates intracellular levels of ROS and MDA, while simultaneously decreasing SOD activity and the GSH/ GSSG ratio, in both in vivo and in vitro. These findings indicate the crucial role of oxidative stress in Inetetamab-induced cardiotoxicity.

Myocardium exhibits a high mitochondrial density, which comprises approximately 40% of the total cardiomyocyte volume. Mitochondria are extremely dynamic organelles that are primarily responsible for ATP synthesis^24^. The balance of mitochondrial dynamics is crucial for ensuring their proper function, which involves regulating various processes such as mitochondrial respiration, metabolism and generation of ROS^25^. It was suggested that disruption of mitochondrial dynamics is associated with the development of cardiovascular disorders, such as ischemia-reperfusion injury and cardiotoxicity induced by anti-cancer drugs^26^. Mitochondria, being the primary source and target of ROS, undergo oxidative DNA damage in response to ROS accumulation, which disrupts the mitochondrial membrane potential, prompting the release of cytochrome C into the cytosol, and subsequently initiates activation of Caspase-9 and the recruitment of additional Caspases, ultimately leading to cell apoptosis^27^. In this study, we speculated that Inetetamab-induced cardiotoxicity may be associated with apoptosis resulting from the disruption of mitochondrial dynamics. We observed that the mitochondrial transmembrane potential in the H9c2 cells was decreased in a dose-dependent manner following Inetetamab administration. Furthermore, based on the results of TUNEL staining, Western blotting and immunochemistry assays, we found that Inetetamab can induce apoptosis in cardiomyocytes, characterized by decreased Bcl-2 expression, increased Caspase-3 activation and elevated Bax expression. This suggests that the mitochondrial pathway may be involved in Inetetamab-induced cardiotoxicity through its participation in the complex interactions between oxidative stress and apoptosis.

Autophagy constitutes a complex physiological process that can be triggered by diverse stimuli, including starvation, oxidative stress and infection, ultimately serving to modulate both cellular and energetic homeostasis^28^. Commonly, autophagy performs a protective role against cell damage, thereby promoting cell survival and human health. However, disruptions in autophagy mechanisms or excessive autophagic activity typically result in cell death^28^. It has been suggested that abnormal autophagy serves as a notable risk factor for patients with cardiac diseases, especially those afflicted with various cardiomyopathies, such as coronary artery disease, hypertension, aortic valvular disease and congestive heart failure^29^. Excessive autophagic activity may be harmful to heart under specific stressful conditions, such as reperfusion injury, resulting in selective degradation of catalase, increased accumulation of ROS, and even cell death^30,31^. In this study, we demonstrated that Inetetamab elicits abnormal autophagy response in cardiomyocytes, manifested by a dose-dependent upregulation of the expressions of autophagy-related proteins P62 and Beclin1. Furthermore, AO staining revealed that Inetetamab treatment markedly augments autophagic activity in H9c2 cells, which is contrary to our cell viability results demonstrated by CCK-8 assay. This suggests that excessive autophagic activity is involved in Inetetamab-induced cardiotoxicity. However, a recent study reported that trastuzumab suppresses autophagy and Beclin 1 expression in cardiomyocytes, which is inconsistent with our results^32^. Numerous studies have documented the relationship between oxidative stress, apoptosis and autophagy^33–35^. For instance, Zhang et al. revealed that oxidative stress-induced granulosa cell (GC) death is a pivotal factor in follicular atresia. Both autophagy and apoptosis, as the primary forms of programmed cell death, have been observed in response to H_2_O_2_-induced oxidative stress and have been implicated in GC death^33^. Building upon these insights, our findings underscore the complex interplay between oxidative stress, apoptosis and autophagy in Inetetamab-induced cardiotoxicity. Notably, Inetetamab-induced oxidative stress likely acts as an upstream signal that initiates apoptosis and autophagy, resulting in cardiomyocyte injury and ultimately contributing to Inetetamab’s cardiotoxic effects.

**Fig. 7:**
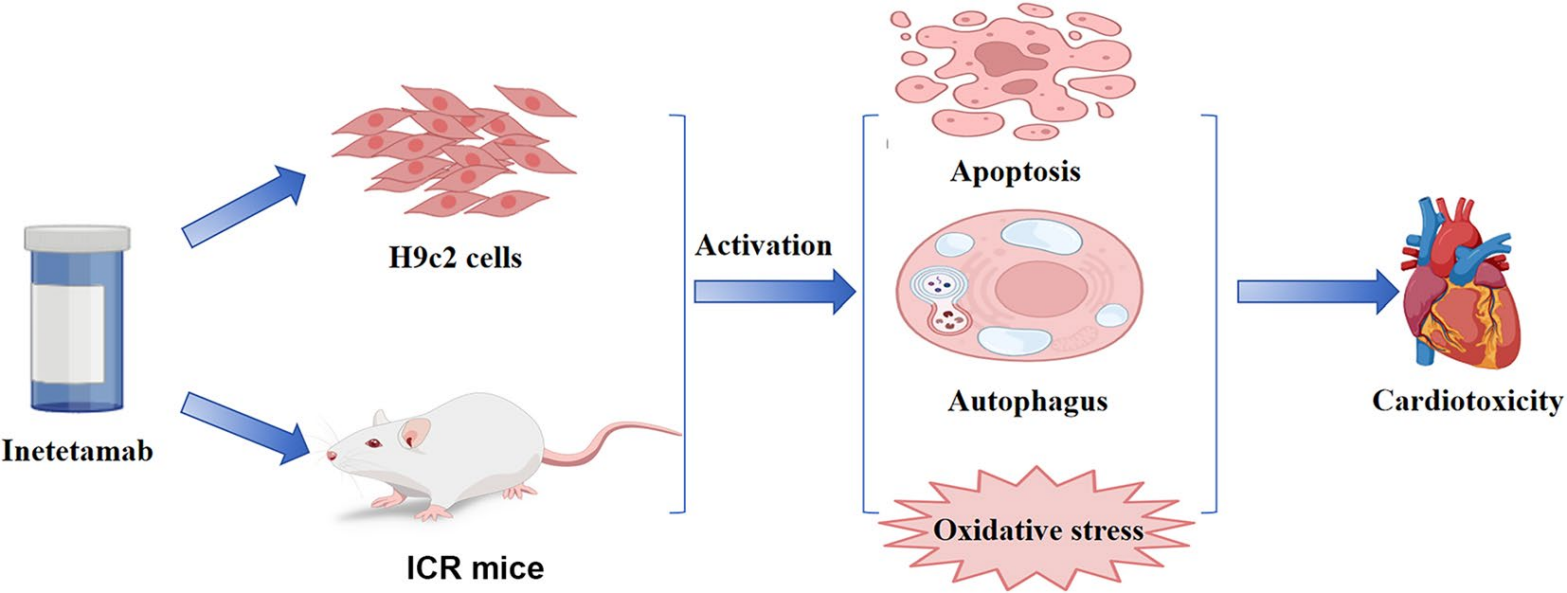
Multilevel and multifactorial mechanisms underlying Inetetamab-induced cardiotoxicity: Involvement of oxidative stress, apoptosis and autophagy. (The figure was generated using Adobe Illustrator 2024, Version 28.0. The software and related resources can be accessed via the following URL: https://www.yuque.com/chuyong-xj4em/gztpn2.).

Monoclonal antibody therapy, including Inetetamab, has been demonstrated to significantly improve outcomes for patients with HER2 + breast cancer. Nevertheless, its clinical utilization is substantially limited by the potential risk of adverse effects, including left ventricular dysfunction and heart failure. Therefore, it is essential to undertake further research to gain a deeper understanding of the mechanisms behind monoclonal antibody-induced cardiotoxicity. In conclusion, the present study suggests that the mechanism underlying Inetetamab-induced cardiotoxicity involves multilevel and multifactorial interactions. Inetetamab, in a dose-dependent manner, activates oxidative stress, apoptosis and autophagy in both cardiac H9c2 cells and ICR animals, ultimately resulting in adverse cardiotoxic effects (Fig. 7).

## Electronic supplementary material

Below is the link to the electronic supplementary material.

Supplementary Material 1

## Data Availability

The datasets generated during and/or analysed during the current study are available from the corresponding author on reasonable request.
