# Supplementary material for: Inetetamab triggers cardiotoxicity through its interaction with apoptosis, oxidative stress and autophagy pathways
Source: Sci Rep. 2025 Jul 1;15:20987. doi: 10.1038/s41598-025-02125-5 (PMC12217453; doi:10.1038/s41598-025-02125-5)

A B C D E F represents western blot analysis shown in Fig 4

lane: control 2.5 4.5 8 (mg/ml)

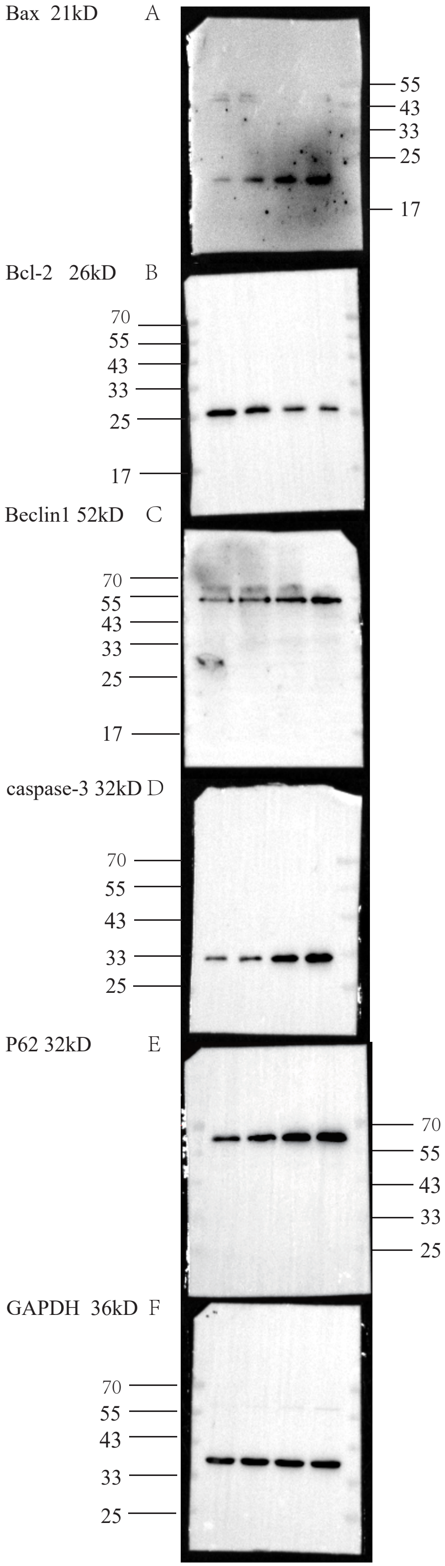

a b c d e f represents western blot analysis shown in Fig 6

lane: control 4 8 (mg/ml)

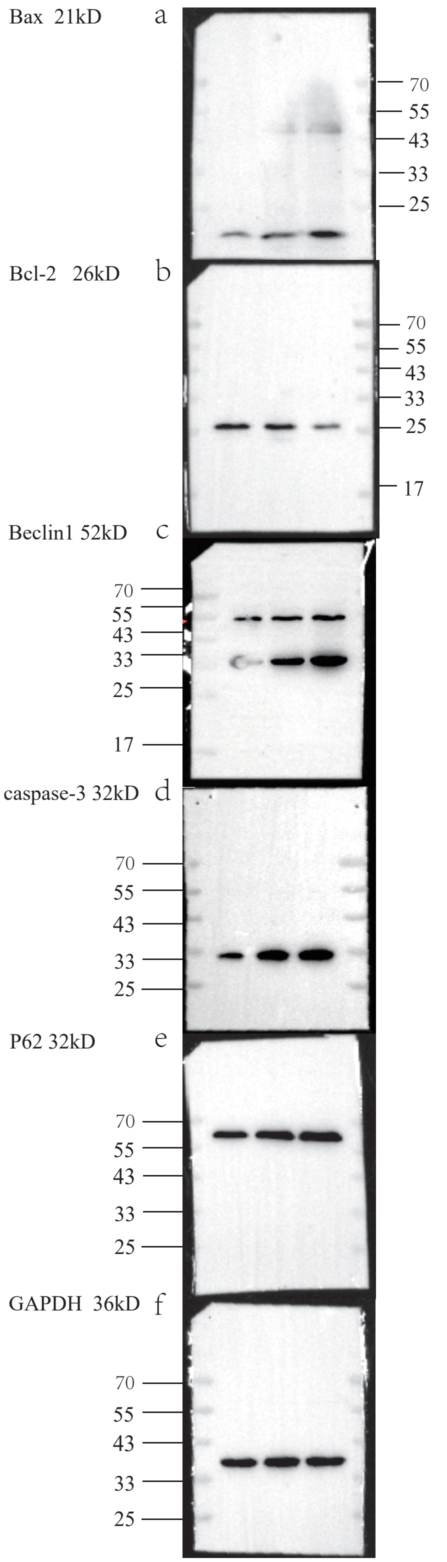

Supplement: Supplementary file 1 — Supplementary Material 1 [file 41598_2025_2125_MOESM1_ESM.pdf]
